# The Effect of Appearance Anxiety on Social Anxiety among College Students: Sequential Mediating Effects of Self-Efficacy and Self-Esteem

**DOI:** 10.3390/bs13080692

**Published:** 2023-08-19

**Authors:** Jieying Liao, Tiansheng Xia, Xuan Xu, Li Pan

**Affiliations:** 1 School of Art and Design, Guangdong University of Technology, Guangzhou 510090, China; 2 Guangdong International Center of Advanced Design, Guangdong University of Technology, Guangzhou 510090, China

**Keywords:** appearance anxiety, self-efficacy, self-esteem, social anxiety, college students

## Abstract

Social anxiety is a serious psychological problem prevalent among Chinese college students, and appearance anxiety plays an important role in its development. Although some studies have explored the relationship between the two, the mediating mechanism is unclear. This study constructed a sequential mediation model based on the cognitive–behavioral theory of body image disorder and social phobia to explore the mediating role of self-efficacy and self-esteem. A total of 234 college students were recruited using the convenience sampling method, including 68 males (29.10%) and 166 females (70.90%), with an average age of 21.25 years (SD = 1.75). Data were collected using the Social Appearance Anxiety Scale, the General Self-Efficacy Scale, the Self-Esteem Scale, and the Interaction Anxiety Scale. The study’s results demonstrated a significant and positive predictive relationship between appearance anxiety and social anxiety (effect = 0.21, SE = 0.04, 95% CI = [0.13, 0.29], *p* < 0.001). Appearance anxiety not only directly affects social anxiety but also has three indirect paths affecting social anxiety: the independent mediating effect of self-efficacy (effect = 0.03, SE = 0.01, 95% CI = [0.00, 0.06], *p* < 0.001), the independent mediating effect of self-esteem (effect = 0.03, SE = 0.02, 95% CI = [0.01, 0.07], *p* < 0.001), and the serial mediation effect of self-efficacy and self-esteem (effect = 0.01, SE = 0.01, 95% CI = [0.00, 0.03], *p* < 0.001). Direct and indirect effects accounted for 73.81% and 26.19% of the total effect, respectively. These findings provide new perspectives on the intervention with and treatment of social anxiety in college students.

## 1. Introduction

Social anxiety, as described by Morrison and Heimberg [1], pertains to an irrational fear that individuals experience during social interactions, stemming from their apprehension of receiving attention and being observed, scrutinized, or evaluated by others. In China, the level of social anxiety among college students has shown a yearly increase [2]. During the period from 2016 to 2020, relevant research found that 22.4~45.7% of college students had different degrees of social anxiety problems [3,4,5]. This implied that an increasing number of Chinese college students suffered from social anxiety. If social anxiety is not mitigated, it may develop into a severe social anxiety disorder. Individuals with social anxiety disorder report low quality of life and severe impairments in social, occupational, and educational functioning [6,7]. In addition, a recent meta-analysis of the relationship between social anxiety and suicide in youth showed that higher levels of social anxiety were associated with more frequent suicidal ideation and higher current suicidal risk [8]. Given the harmfulness of social anxiety, it is necessary to investigate its mechanism and formulate timely intervention measures for college students with social anxiety.

Previous research has found that appearance anxiety, a unique construct highly associated with social anxiety, is one of the important risk factors for social anxiety in college students [9,10]. Individuals who are dissatisfied with their appearance may be more introverted, pessimistic, insecure, and uncomfortable in social situations [11,12,13]. More direct evidence shows that dissatisfaction with appearance can significantly affect social interactions [14] and increase social anxiety [15]. Despite scholars discussing the association between appearance anxiety and social anxiety among college students, the underlying mechanism that connects the two remains unclear. Research on this issue is beneficial for the alleviation of anxiety in college students. Research has suggested that cognitive factors may play a role in the etiology or maintenance of social anxiety [1]. Individuals with social anxiety exhibit dysfunctional thoughts and beliefs that lead to anxiety and avoidance, and they perceive the environment as threatening and dangerous [1]. When individuals are overly self-focused in a particular area, they develop a fear of negative evaluations from others, triggering dysfunctional beliefs that produce negative self-evaluations and negative emotions. Individuals often resort to social avoidance behaviors as a protective mechanism to alleviate negative emotions or to prevent harm from negative evaluations. These thoughts and behaviors also reinforce an individual’s anxiety, creating a vicious cycle. Based on past research on the mechanisms of social anxiety, it was found that when individuals overfocus on their self-image, the anxiety generated by that self-image may cause reduced self-efficacy and low self-esteem [16] and further trigger social anxiety. Therefore, it is reasonable to hypothesize that self-efficacy and self-esteem may mediate the relationship between appearance anxiety and social anxiety.

### 1.1. Appearance Anxiety and Social Anxiety

Appearance anxiety has been defined as a preoccupation with one’s appearance and a fear that one’s appearance (body and face shape, height, and weight) may be negatively evaluated by others [9]. Appearance anxiety is one of the subclinical indicators of a body anxiety disorder [17], an intrusive psychological state characterized by preoccupation with actual or perceived deficits in appearance and repetitive behaviors such as checking, grooming, and comparing oneself to others to cope with these concerns [18]. College students are a high prevalence group for appearance anxiety, and related studies have shown that the prevalence of self-reported appearance anxiety among college students abroad ranges from 1.20 to 13.00% [19], while a domestic survey geared toward appearance anxiety among college students found that the prevalence of appearance anxiety among college students in China was as high as 59.03% [20]. Appearance anxiety is known to be one of the important factors contributing to social dysfunction, which may lead to severe interpersonal and psychological distress [21,22]. Studies have found that appearance anxiety is significantly associated with many psychological problems and self-evaluation disorders, and it is a significant predictor of social anxiety [23,24,25]. Clark and Wells’ [26] cognitive model of social phobia posited that negative cognitive beliefs predispose socially anxious individuals to perceive the social environment as threatening or dangerous. One of the important factors in maintaining social anxiety is self-focused attention (i.e., monitoring self-perceptions and predicting negative evaluations from others). According to the cognitive model of social phobia, appearance anxiety is a result of individuals turning their self-focused attention to their image and experiencing fears that their appearance may be negatively evaluated by others, and these behaviors typically increase the severity of social anxiety. Accordingly, this study proposed hypothesis H1: appearance anxiety positively predicts the onset of social anxiety.

### 1.2. The Mediating Effect of Self-Efficacy

Self-efficacy is defined as an individual’s belief in their ability to control their functioning and the events that affect their life [27]. Bandura [28] viewed perceived self-efficacy as an individual’s belief in their ability to master specific activities, situations, or aspects of their own psychological and social functioning. From this perspective, self-efficacy is viewed as domain-specific. Previous research has shown that people with social anxiety tend to have lower self-efficacy [29,30]. Leary and Atherton [31] clarified the link between self-efficacy and social anxiety in adults by introducing the concept of “self-efficacy expectations of performance”. On the other hand, according to social cognitive theory, beliefs about personal efficacy influence health behaviors both directly and through their facilitators or barriers to goals, outcome expectations, and perceptions [32]. That is, people with low self-efficacy will develop negative perceptions about their social behavior, believing that they are failing in their performance, and even though this is not the case, they tend to engage in social avoidance behaviors and develop a fear of socializing to avoid negative evaluations or to reduce anxiety.

Although there is no research directly showing the relationship between appearance anxiety and self-efficacy, several studies have shown that dissatisfaction with body image can lead to maladaptive consequences for one’s physical and mental health, including depression, anxiety, low self-esteem, and eating disorders [33,34,35]. This is in line with social cognitive theory and indirectly suggests that appearance anxiety leads to lower self-efficacy. Furthermore, empirical studies have also shown that self-efficacy mediates between negative self-evaluation and social anxiety [36]. Individuals experiencing appearance anxiety generally exhibit negative self-evaluations regarding their appearance. Accordingly, this study proposed hypothesis H2: self-efficacy plays a mediating role in the relationship between appearance anxiety and social anxiety.

### 1.3. The Mediating Effect of Self-Esteem

Self-esteem has traditionally been defined as an overall positive or negative self-evaluation [37]. However, some researchers had advocated focusing on the specific domains on which self-esteem is based [38,39]. According to this view, people’s overall self-worth depends on success or failure in one or more specific contingent domains. That is, when people base their self-worth on their appearance, it can have a specific negative impact not only on people’s perceptions and satisfaction with their appearance but also on their overall self-evaluation. Appearance-based self-worth is significantly negatively related to self-esteem compared to other contingent domains [40,41]. Furthermore, it has been shown that the higher a person’s dissatisfaction with their appearance, the worse their perception of their overall sense of self-worth [42,43], and this is typically measured based on self-esteem [37,44]. On the other hand, lower self-esteem is strongly associated with social anxiety [45,46], and self-esteem is a risk factor for social anxiety [47]. In their study exploring social anxiety among a sample of undergraduate students, Kocovski and Endler [48] found that low self-esteem was a predictor of heightened fear of negative evaluations, which subsequently led to an increase in social anxiety.

Numerous studies have shown that self-efficacy is closely related to self-esteem [49,50]. Self-efficacy and self-esteem are two important and similar psychological concepts, but they refer to two different dimensions: self-acceptance and self-evaluation, respectively. Self-esteem involves a stable assessment of one’s self-worth [51], whereas self-efficacy refers to individuals’ judgments about their abilities [52]. Bandura suggested that self-efficacious beliefs may contribute to the motivation and effort required to achieve desired goals, which may promote one’s self-esteem [52]. Similarly, when individuals have lower self-efficacious beliefs, this can also lead to lower self-esteem. Since there is no fixed relationship between self-efficacy and self-esteem, but self-esteem reflects a stable evaluation, perceived self-efficacy may change significantly across situations and time. Thus, the relationship between self-esteem and self-efficacious beliefs depends on the type of self-efficacious belief under consideration [53], such as appearance attractiveness. It can be hypothesized that self-efficacy may produce significant changes in self-competence judgments when it is influenced by appearance anxiety, which, in turn, may affect the overall evaluation of oneself and lead to the occurrence of social anxiety. Accordingly, the present study proposed hypothesis H3: appearance anxiety affects social anxiety not only through the independent mediating role of self-esteem but also through the sequential mediating role of self-efficacy and self-esteem.

### 1.4. Research Purposes

**Figure 1:**
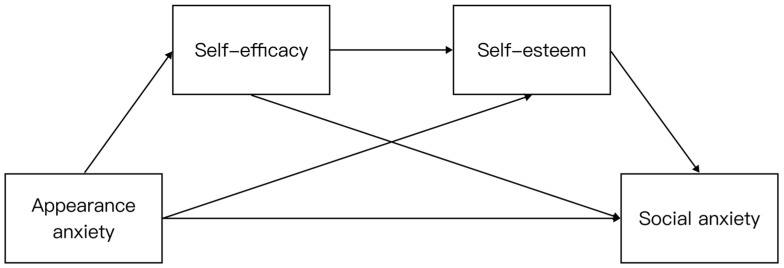
A predictive model of social anxiety in college students.

In summary, we developed a serial mediation model (Figure 1). Drawing on insights from prior studies, the purpose of the research mainly includes three aspects: H1: appearance anxiety exerts a significant positive effect on social anxiety; H2: self-efficacy mediates the relationship between appearance anxiety and social anxiety; H3: appearance anxiety affects social anxiety not only through the independent mediating role of self-esteem but also through the serial mediating role of self-efficacy and self-esteem.

## 2. Method

### 2.1. Participants and Procedure

The participants in this study constituted a convenience sample of 234 students aged between 18 and 25, selected from the Guangdong University of Technology. We used a professional survey platform “Questionnaire Star” (https://www.wjx.cn/; accessed on 8 November 2022) to publish the questionnaire, and we sent it to the participants via WeChat and email. The participants completed the survey online. Data for the study were collected from 9 to 12 November 2022. This study was approved by the Ethics and Academic Committee of the Guangdong University of Technology (No. GDUTXS2023124) before data collection. In addition, participants signed written informed consent before completing the online questionnaire and were informed that they could ask questions or withdraw from the assessment at any time and that all data would be kept strictly confidential.

### 2.2. Measures

#### 2.2.1. Social Appearance Anxiety Scale

The Chinese version of the Social Appearance Anxiety Scale was used to examine the overall level of appearance anxiety among college students [9,54], which has good reliability and applicability in studies of Chinese student populations [55]. The scale uses a 5-point scale (1 = strongly disagree, 5 = strongly agree) and has a total of 16 items, such as “I get tense when it is obvious people are looking at me”. The internal consistency coefficient of the scale in this study was 0.94.

#### 2.2.2. General Self-Efficacy Scale

The General Self-Efficacy Scale (GSES) was used to measure the degree of self-efficacy of college students. The scale was developed by German scholars Schwarzer and Jerusalem [56], and the Chinese version of the GSES was translated and revised by Wang and Cai-Kang et al. [57], with good reliability and validity. A score below 2.50 would indicate that the subject’s self-efficacy was at a low level, and a higher total score would indicate higher self-efficacy. The internal consistency coefficient of the scale in this study was 0.92.

#### 2.2.3. Self-Esteem Scale

Next, we measured using the Self-Esteem Scale [37]; the Chinese version was translated and revised by Tian [58] and has been widely used in studies of Chinese student populations with good reliability and validity [59,60]. The Self-Esteem Scale consists of 10 items and is scored using a four-point Likert scale (“strongly disagree” to “strongly agree”). The internal consistency coefficient of the scale in this study was 0.87.

#### 2.2.4. Social Anxiety Scale

The social anxiety level of college students was measured using the Social Anxiety Scale, which was compiled by Peng and Gong [61]. The scale includes 15 items, such as “I usually feel nervous when calling someone I don’t know very well”, of which the 3rd, 6th, 10th, and 15th questions are reverse scoring questions. The scale uses a 5-point scale (“strongly disagree” to “strongly agree”). The internal consistency coefficient of the scale in this study was 0.84.

### 2.3. Analytical Method

Statistics were generated and data analysis was performed using SPSS 26.0. First, this study tested for common method bias using exploratory factor analysis. Then, descriptive statistics and correlation analysis were used to calculate the mean, standard deviation, and correlation coefficients of all variables, and independent-samples t-tests were used to determine gender differences in the variables. Finally, to test the mediated effects, we employed the bias-corrected nonparametric percentile bootstrap method, as recommended by Wen and Ye [62]. We utilized Hayes’ [63] PROCESS macro model 6 to identify the serial mediated effects, which involved bootstrapping with 5000 samples to obtain a 95% confidence interval.

### 2.4. Common Method Biases Test

Exploratory factor analysis was used to test for possible common method biases [64]. The findings revealed that 11 factors in total possessed eigen root values exceeding 1. Additionally, the first common factor accounted for only 20.32% of the overall variance, falling short of 40.00%. This finding suggests that the data in this study are not affected by significant common method bias.

## 3. Results

### 3.1. Descriptive Statistics and Correlation Analysis

Descriptive statistics of the age and gender of the participants (Table 1) showed that of the 234 students sampled, 68 (29.10%) were males and 166 (70.90%) were females. The mean age of the sample was 21.25 years (SD = 1.75). An independent-samples t-test was used to explore the differences between genders in appearance anxiety, self-efficacy, self-esteem and social anxiety. The results, as shown in Table 2, showed that there was no significant difference between genders on the variables (*p* > 0.05), indicating that the variables were not affected by gender.

The Kolmogorov–Smirnov (K-S) test was conducted as a preliminary normality test, and the results showed that the *p*-value of the variables was bigger than 0.05. Utilizing a Pearson correlation analysis, potential associations among appearance anxiety, self-efficacy, self-esteem, and social anxiety were examined. The descriptive statistics for each variable can be found in Table 3. Appearance anxiety exhibited significant positive correlations with social anxiety and significant negative correlations with self-efficacy and self-esteem. Self-efficacy displayed significant positive correlations with self-esteem and significant negative correlations with social anxiety. Furthermore, self-esteem showed a significant negative correlation with social anxiety.

### 3.2. Mediating Effect Test

To ensure the accuracy of the results, we set gender as a covariate in the structural equation modeling. The regression analysis results (Table 4) showed that appearance anxiety significantly negatively predicted self-efficacy (*β* = −0.16, *p* < 0.001), appearance anxiety significantly negatively predicted self-esteem (*β* = −0.13, *p* < 0.001), and self-efficacy significantly positively predicted self-esteem (*β* = 0.36, *p* < 0.001). When considering all the variables simultaneously in the regression equation, the following effects were observed: Appearance anxiety exhibited a significant and direct predictive effect on social anxiety (*β* = 0.21, *p* < 0.001). Self-efficacy, on the other hand, significantly and negatively predicted social anxiety (*β* = −0.17, *p* < 0.05). Similarly, self-esteem displayed a significant and negative predictive relationship with social anxiety (*β* = −0.26, *p* < 0.001).

**Figure 2:**
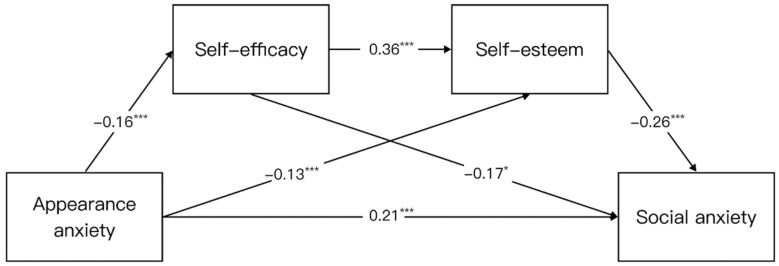
The final model illustrates the chain mediation of the associations between self-efficacy and self-esteem affecting appearance anxiety and social anxiety. Note: * *p* < 0.05, *** *p* < 0.001.

In the mediation model, a total of 1 direct effect path and 3 mediating effect paths were formed (Table 5 and Figure 2). The direct effect (0.21) and indirect effect (0.07) accounted for 73.81% and 26.19% of the total effect (0.28), respectively. Among them, the indirect effect value of path 2 is 0.03, accounting for 9.35% of the total effect (SE = 0.01, 95% CI = [0.00, 0.06], *p* < 0.001); the indirect effect value of path 3 is 0.03, accounting for 11.76% of the total effect (SE = 0.02, 95% CI = [0.01, 0.07], *p* < 0.001); the indirect effect value of path 4 is 0.01, accounting for 5.04% of the total effect (SE = 0.01, 95% CI = [0.00, 0.03], *p* < 0.001).

## 4. Discussion

This study examined the roles of both self-efficacy and self-esteem in the relationship between appearance anxiety and social anxiety among college students, and it explained how appearance anxiety affects social anxiety by influencing cognitive factors in college students. Thus, this study provided a theoretical integration to better explain the internal mechanisms through which appearance anxiety affects social anxiety, and it provided theoretical guidance for mitigating the negative effects of social anxiety in college students. This study showed that appearance anxiety not only affects social anxiety directly but also indirectly through the mediating roles of self-efficacy and self-esteem. This mediating effect is generated in three ways: first, through the independent effect of self-efficacy; second, through the independent effect of self-esteem; and third, through the joint effects of self-efficacy and self-esteem.

### 4.1. The Impact of Appearance Anxiety on Social Anxiety among College Students

The current investigation revealed a notable positive correlation between appearance anxiety and social anxiety among college students, aligning with previous research [9,10]. The results of this study validated the cognitive–behavioral model of body dysmorphic disorder [65] and the cognitive model of social phobia [26], that is, when self-consciousness is focused on appearance images, it will increase the fear of negative evaluations. To avoid risk factors in fearful situations, appearance-anxious individuals adopt avoidance or conformity as safety behaviors, which manifest as social avoidance behaviors in social contexts. In turn, there is a negative feedback loop for such safety behaviors that can exacerbate an individual’s social anxiety. Furthermore, associations between social anxiety and maladaptive appearance image and body image disorders have been found in clinical samples [66,67,68,69].

Different scholars have offered different perspectives on the explanation of the correlation between appearance anxiety and social anxiety. Some scholars have suggested that appearance anxiety may also be triggered by social anxiety [70], while others have argued that fear of negative evaluations mediates the link between appearance anxiety and social anxiety [71]. Additionally, previous studies have found that self-compassion [72], perfectionism [73], and social rejection sensitivity [21] are also present and work to produce a relationship between the two. However, no studies have been found to longitudinally examine the causal relationship between appearance anxiety and social anxiety, and these are a focus for future research.

### 4.2. The Role of Self-Efficacy as a Mediator

The current study established that self-efficacy served as a mediator in the relationship between appearance anxiety and social anxiety and that appearance anxiety decreased self-efficacy by increasing patients’ dysfunctional beliefs, thereby increasing the likelihood of social anxiety, which was a finding that was consistent with previous research that found that appearance anxiety leads to low self-efficacy [33,34] and low self-efficacy induces social anxiety [31]. The present study simultaneously included all three variables in the examination, demonstrating that self-efficacy plays an important mediating role in the process by which appearance anxiety influences socially anxious behavior. The social cognitive theory of self-efficacy posits that behavior is strongly stimulated by self-influence [27]. College students who suffer from appearance anxiety have lower associated levels of self-efficacy, and low self-efficacy may increase an individual’s reliance on dysfunctional coping strategies to cope with anxiety in social situations [30]. In turn, these dysfunctional coping strategies may exacerbate the disabling experience of social anxiety.

### 4.3. The Role of Self-Esteem as a Mediator

This study established that self-esteem served as a mediator in the relationship between appearance anxiety and social anxiety in college students, and that appearance anxiety led to social anxiety by lowering an individual’s overall self-evaluation. In other words, self-esteem is one of the important mechanisms through which appearance anxiety affects social anxiety among college students. This study provided a possible explanation for the mechanism of how appearance anxiety affects social anxiety in college students from the perspective of the internalization of self-perceptions. According to the cognitive model of social phobia [1,74], negative self-views play important roles in the maintenance of the disorder. Particularly, holding negative self-views can result in disparities between one’s self-perceptions and the standards set by others, which, in turn, leads to heightened fear of negative evaluations. When faced with social threats, individuals tend to direct their attention inwards and engage in meticulous self-monitoring processes. During such self-monitoring, they experience excessively negative self-images and perceive these self-images as accurate representations of themselves [16]. Therefore, college students who possess appearance anxiety are prone to basing their self-worth on their appearance, which negatively affects their overall self-evaluation, and a negative self-view can further trigger social anxiety.

### 4.4. The Sequential Mediation Effect of Self-Efficacy and Self-Esteem

The current investigation additionally revealed that self-efficacy and self-esteem act as sequential mediators between appearance anxiety and social anxiety, affirming the robust connection between self-efficacy and self-esteem, and supporting prior research [49,75]. The findings indicated that appearance anxiety raises the likelihood of social anxiety, with this influence being influenced by a combination of self-acceptance (self-efficacy) and self-evaluation (self-esteem). Self-efficacy and self-esteem, as major components of the self-system, are what guide the development of individual behavior to meet new demands and challenges in the environment [53]. Self-efficacious beliefs are seen as psychological constructs that contribute significantly to the development and promotion of optimal levels of self-esteem [76,77]. This also reveals that self-efficacy and self-esteem are risk and maintenance factors that influence individual anxiety. Externally anxious individuals develop negative self-efficacious beliefs due to an extreme lack of confidence in their image and form false self-worth judgments in the ego system, which negatively affects the individuals’ self-esteem levels and contributes to their inability to adapt to social environments and interpersonal challenges, which triggers social anxiety.

In the analysis of studies about the mediating effect of social anxiety, the effect values were found to range from 7.18 to 37% [78,79], both of which were considered to provide new intervention strategies for predicting college students’ social behaviors and ameliorating social anxiety. The mediating effect accounted for less in the present study, which is similar to the results of recent effect analyses on the effect of self-compassion-mediated appearance anxiety on social anxiety [72], but it also confirms the theoretical validity of the theoretical model, which implies that self-efficacy and self-esteem represent a potential mechanism of action that can partially explain the relationship between appearance anxiety and social anxiety. In other words, increasing self-efficacy and self-esteem as a way to improve self-perception may help alleviate social anxiety among college students.

### 4.5. Implications and Limitations

This study revealed the influence of appearance anxiety on social anxiety and the mechanisms underlying the role of self-efficacy and self-esteem in generating both, providing a theoretical basis for effective interventions for social anxiety among college students while generating important implications for the development of students’ mental health during this period. The results of the study revealed that college students’ self-esteem and self-efficacy are susceptible to decreases due to appearance anxiety, which, in turn, causes decreases in social competence and triggers social anxiety. The study by von Soest et al. [80] also showed that appearance is the most important determinant of self-esteem in adolescence and youth. Therefore, educational institutions and related organizations should aid students in cultivating a healthy self-concept, promoting varied aesthetic values, and embracing diverse perspectives on aesthetics. Only when individuals focus on the perceptions and realizations about their intrinsic self-worth and explore their diverse beauty can they effectively enhance their self-confidence in interpersonal interactions and avoid the occurrence of social anxiety.

In addition, the present study had some limitations that need to be improved in future research: first, the study used a cross-sectional study design to explore appearance anxiety, social anxiety, and their underlying mechanisms, and it excluded conclusions about causal relationships between the variables [81]. Second, all data were collected via self-report scales, which may have influenced the results due to respondent bias and recall bias. Third, the study participants were all from the same university, and the gender ratio in the study was not balanced. Future studies may consider expanding the sample to balance the gender ratio. Finally, there are many factors influencing social anxiety among college students, and future studies may consider including more variables, such as social comparison, cyberbullying victimization, and adult attachment, which are closely related to social anxiety [78,82,83], to conduct in-depth explorations of college students’ social anxiety and thereby provide insights into the formation mechanisms of social anxiety among college students, improve the mediation effect percentage of the mediation model, and offer new insights that will improve the physical and mental health of college students.

## 5. Conclusions

The results of this study indicated that appearance anxiety was a significant contributing factor to social anxiety among college students, and their relationship was mediated by self-efficacy and self-esteem. Guiding college students to establish correct self-cognition and aesthetic value was beneficial to reduce social anxiety.

## Figures and Tables

**Table 1:** Socio-demographic characteristics of the study sample (*n* = 234).

Variables		N/M	%/SD
Age		21.25	1.75
Gender	Male	68	29.10
	Female	166	70.90

Note: M: mean. SD: standard deviation. The same notes apply to Table 2 below.

**Table 2:** T-test results for gender in each variable (*n* = 234).

	Gender	N	M	SD	t	*p*
1. Appearance Anxiety	Male	68	2.88	0.77	−0.32	0.75
	Female	166	2.92	0.84		
2. Self-efficacy	Male	68	2.74	0.41	0.46	0.65
	Female	166	2.71	0.45		
3. Self-esteem	Male	68	2.88	0.44	0.62	0.54
	Female	166	2.84	0.44		
4. Social anxiety	Male	68	3.30	0.58	−1.49	0.14
	Female	166	3.42	0.52		

**Table 3:** Mean, standard deviation, and the correlation matrix of each variable (*n* = 234).

	M	SD	1	2	3	4
1. Appearance anxiety	2.91	0.82	-			
2. Self-efficacy	2.85	0.44	−0.29 **	-		
3. Self-esteem	2.72	0.44	−0.34 **	0.43 **	-	
4. Social anxiety	3.38	0.54	0.43 **	−0.32 **	−0.38 **	-

Note: ** *p* < 0.01. The same description applies to the table below. M: mean. SD: standard deviation.

**Table 4:** Mediating effects of self-efficacy and self-esteem on the relationship between appearance anxiety and social anxiety.

	Self-Efficacy			Self-Esteem			Social Anxiety		
	b	SE	t	b	SE	t	b	SE	t
Constant	3.21	0.14	22.23 ***	2.30	0.23	9.79 ***	3.82	0.34	11.37 ***
Gender	−0.02	0.06	−0.38	−0.02	0.06	−0.43	0.09	0.07	1.38
Appearance anxiety	−0.16	0.03	−4.60 ***	−0.13	0.03	−3.97	0.21	0.04	5.22 ***
Self-efficacy				0.36	0.06	5.88 ***	−0.17	0.08	−2.21 *
Self-esteem							−0.26	0.08	−3.30 ***
R^2^	0.08			0.23			0.27		
F	10.68			23.37			21.39		

Note: * *p* < 0.05, *** *p* < 0.001. SE: standard error.

**Table 5:** Testing the mediation effect of appearance anxiety on social anxiety.

	Effect	BootSE	BootLLCI	BootULCI	Ratio of Total Effects
Direct Effect					
Path 1: Appearance Anxiety → Social Anxiety	0.21	0.04	0.13	0.29	73.81%
Indirect Effect					
Path 2: Appearance Anxiety → Self-Efficacy → Social Anxiety	0.03	0.01	0.00	0.06	9.35%
Path 3: Appearance Anxiety → Self-Esteem → Social Anxiety	0.03	0.02	0.01	0.07	11.76%
Path 4: Appearance Anxiety → Self-Efficacy → Self-Esteem → Social Anxiety	0.01	0.01	0.00	0.03	5.04%
TOTAL Mediation Effect	0.07	0.02	0.04	0.12	26.19%

Note: BootSE: bootstrap standard error. BootLLCI: bootstrap lower-limit confidence interval. BootUULCI: bootstrap upper-limit confidence interval.

## Data Availability

The data presented in this study are available on request from the corresponding author.
